# Integration of phospholipid-complex nanocarrier assembly with endogenous *N*-oleoylethanolamine for efficient stroke therapy

**DOI:** 10.1186/s12951-019-0442-x

**Published:** 2019-01-19

**Authors:** Xiangrui Yang, Lanxi Xu, Juan Zhou, Yunlong Ge, Shichao Wu, Junxiong Huang, Ying Li, Maoshu Zhu, Xin Jin, Lichao Yang

**Affiliations:** 1 Department of Basic Medical Science, Medical College, Xiamen University, Xiamen, 361102 China; 2 Department of Obstetrics and Gynecology, The First Affiliated Hospital of Xiamen University, Xiamen, 361005 China; 3 Department of Chemistry, College of Chemistry and Chemical Engineering, Xiamen University, Xiamen, 361005 China; 4 Department of Pharmacy, Xiamen Medical College, Xiamen, 361005 China; 5 The Central Laboratory of Fifth Hospital of Xiamen, Xiamen, 361005 China

**Keywords:** *N*-Oleoylethanolamine, Phospholipid, Stroke therapy, Drug delivery

## Abstract

**Background:**

Leading to more and more deaths and disabilities, stroke has become a serious threat to human health. What’s more, few effective drugs are available in clinic till now.

**Results:**

In this research, we prepared a novel neuroprotective nanoformation (OEA–SPC NPs) via the combination of the nanoparticle drug delivery system with the endogenous *N*-oleoylethanolamine (OEA). By forming hydrogen bond between OEA and the carrier—soybean phosphatidylcholine (SPC), the form of OEA was turned into amorphus state when loading to the nanoparticles, which greatly improved its bioavailability. Then the following systematic experiments revealed the efficient neuroprotective effect of OEA–SPC NPs in vivo. Compared with the MCAO group, the cerebral infarct volume was reduced by 81.1%, and the edema degree by 78.4% via the oral administration of OEA–SPC NPs. And the neurological deficit scores illustrated that the MCAO rats treated with OEA–SPC NPs exhibited significantly less neurological dysfunction. The Morris water maze test indicated that the spatial learning and memory of cerebral ischemia model rats were almost recovered to the normal level. Besides, the OEA–SPC NPs could inhibit the inflammation of reperfusion to a very slight level.

**Conclusions:**

These results suggest that the OEA–SPC NPs have a great chance to be a potential anti-stroke formation for clinic application and actually bring hope to thousands of stroke patients.

**Electronic supplementary material:**

The online version of this article (10.1186/s12951-019-0442-x) contains supplementary material, which is available to authorized users.

## Background

Given that there were more than 130,000 deaths each year, stroke has been the fifth leading cause of death in the United States [1]. In addition to the high mortality, the high rate of long-term disability is the other reason which makes stroke a serious threat to human health [2]. What’s worse, the incidence is increasing and the therapeutic option is quite limited [3, 4].

Ischemic stroke represents a series of pathological reactions among the blood microvessel parenchymal cells, which would lead to the injury of parenchymal cells [5, 6]. The pathological process of stroke is a complex and highly dynamic process that includes the interactions between cerebral endothelial cells, the basal lamina, pericytes, astrocytes, microglia and neurons [7]. Although the development of neuroprotective drugs has received considerable research interest and a number of promising neuroprotective agents have been identified in preclinical studies, no one can achieve success in clinical trials [8]. The reason for this are various: (1) the difference between animals and human beings; (2) the individual condition of patients; (3) the complexity of ischemic stroke; (4) the treatment time windows; (5) the serious side effects; (6) the effective dose and course of treatment; and so on.

When the research of neuroprotective drugs have not achieved progress for a long time, our team found that *N*-oleoylethanolane (OEA), an endogenous peroxisome proliferator-activated receptor alpha (PPAR-α) agonist, can protect against acute and chronic ischemic injury and improve spatial cognitive deficits in mice [9–11]. The mechanism is that the deficiency of PPAR-α expression in the brain would aggravate the brain injury after ischemic stroke [12], and the activation of PPAR-α would perform protective effects against brain ischemic injury [13–15]. Apart from other synthetic neuro-protectors, OEA is a naturally occurring ethanolamide lipid, which would address many bottlenecks in the previous clinical trials, such as the difference between animals and humans, the side effect. And then owing to the safety of OEA, the problem of its effective dose and the treatment time windows would be solved easier. Therefore, OEA is a promising neuro-protector which may gain success in clinical trials.

However, OEA is a water-insoluble molecule with a fatty acid-based structure, which would become a major barrier to its absorption [16, 17]. In other words, OEA could not be used in clinic in its current form [18]. The problem of poor solubility comes to be a common difficulty to the new drugs’ R&D [19]. Up to 40% of the new chemical entities in development have been suggested to be “poorly water-soluble” [20]. Whatever their therapeutic effect is, they are often dismissed at the beginning of the pharmacological characterization, just owing to the difficulties of their administration [21]. Nevertheless, given the failure of the neuro-protectors and the pronounced neuroprotective effect of OEA, it’s a real pity to give up developing OEA to be a clinic neuro-protector. Fortunately, the progress in nanotechnology research and development gives OEA another opportunity to enter the clinic [22–24]. As the nanoparticle drug delivery system (NDDS) could greatly improve the solubility, and then enhance the bioavailability, this would make up for the deficiency of OEA’s poor solubility [25, 26]. Significantly, since the application of the NDDS is mostly focused on the treatment or diagnosis of cancer [27–29], the combination of NDDS with stroke therapy might come to an unexpected effect.

Hence, in this work, we combined OEA with the SPC-based nanoparticles. By means of XRD, FT-IR and H^1^NMR, we confirmed that OEA could form hydrogen bonds with SPC, which totally changed the form in OEA–SPC NPs and increased the drug loading efficiency. Possessing a drug loading of 18.3 ± 1.3%, a PDI of 0.181 ± 0.019, a zeta potential of − 16.8 ± 0.9 mv, and a size of 230.7 ± 7.9 nm, OEA–SPC NPs were well-distributed and stable enough for oral administration. Then the in vivo neuroprotective effect were systemic evaluated. At the dosage of 10 mg/kg, with which there were no obvious effects in our previous work [9–11], the OEA–SPC NPs could greatly optimize the damaged motor function, the learning and memory disorder induced by ischemia reperfusion. And they could reduce the cerebral infarct volume reduced by 81.1%, and the edema degree by 78.4%. Significantly, the OEA–SPC NPs could inhibit the inflammation of reperfusion to a very slight level and protect the neurons. With an outstanding neuroprotective effect, the OEA–SPC NPs would become a promising anti-stroke drug delivery system for the clinic application.

## Materials and methods

### Materials

All chemical reagents were of analytical grade and used without further purification unless otherwise stated. OEA (purity grade > 95.0%) and Cy 5.5-NHS were obtained from Sigma Chemical Corp (St. Louis, MO, USA). SPC (soybean phosphatidylcholine, purity grade > 90%) was purchased from Lipoid GmbH (Ludwigshafen, Germany). Dichloromethane (DCM), dimethyl sulfoxide (DMSO), and tetrahydrofuran (THF) were provided by Sinopharm Chemical Reagent Co., Ltd. (Shanghai, China).

### Preparation and characterization of OEA–SPC complex (OEA–SPC)

The OEA–SPC was prepared by a solvent evaporation method. Briefly, 6 mg of OEA powder and 30 mg of SPC were codissolved in a glass pressure vessel with 15 mL of THF, accompanied by vigorous agitation at 40 °C for 10 h. Then, THF was removed via vacuum rotary evaporation with a rotary evaporator (N-1001S-W; EYELA, Tokyo, Japan). The physical mixture of OEA and SPC (OEA & SPC) was prepared at the same weight ratio by grinding them with an agate mortar.

### Preparation of OEA–SPC nanoparticles (OEA–SPC NPs)

The OEA–SPC NPs was synthesized by a nanoprecipitation technique. In brief, 10 mL of DCM was added to the OEA–SPC (6 mg of OEA), and then the clear homogeneous solution was then dropwise (0.2 mL/min) introduced into 40 mL of distilled water under magnetic stirring (200 rpm/min). Subsequently, the dispersed phase gradually evaporated with stirring overnight to remove the DCM, producing a clear suspension and resulting in the formation of the OEA–SPC NPs.

### Characterization

The OEA–SPC NPs was analysed using XRD (Phillips X’pert Pro Super), FTIR (Bruker IFS-55 FTIR spectrometer), and H^1^NMR (AVANCE III 600 MHz). The bulk OEA powers, SPC, and the physical mixture of OEA and SPC were used as control. Morphology of the OEA–SPC NPs was examined by SEM (UV-70) and TEM (JEM-2100) at 5 and 200 kV, respectively. The Size and zeta-potential values were determined by a Malvern Zetasizer Nano-ZS machine (Malvern Instruments, Malvern). Three parallel measurements were carried out to determine the average values. The content of OEA in OEA–SPC NPs was determined by LC–MS (3200Qtrap). The content efficiency was calculated by Eq. (1):

1 $${\text{Drug loading content of OEA}}\left( {{\% }} \right) = ( {\text{weight of OEA in NPs}})/( {\text{weight of NPs}}) \times 100{{\%}}$$

### In vitro drug release study

The in vitro drug release studies of OEA–SPC NPs were performed via the dialysis technique. The bulk OEA powers and OEA–SPC NPs were dispersed in a PBS buffer solution (12 mL) and placed in a pre-swelled dialysis bag (MWCO 3500 Da). Then, the dialysis bag was then immersed in PBS (0.1 M, 150 mL, pH 7.4) and oscillated continuously in a shaker incubator (180 rpm) at 37 °C. All samples were assayed by LC–MS.

### Biodistribution

For in vivo fluorescence imaging, Cy 5.5 was conjugated to OEA. Briefly, 1 g of OEA was dissolved in 2 mL of DMSO, and then added with 100 µL Cy 5.5-NHS, accompanied by agitation at rt for 4 h. Then, the suspension was added with 10 mL of DI water and dialyzed against DI water to remove excess Cy 5.5 molecules. The remaining suspension was centrifuged (5000 rpm) and lyophilized for 24 h to obtain the dry OEA-Cy 5.5 powder. OEA-Cy 5.5 and OEA (-Cy 5.5)-SPC NPs ([OEA] = 1 mg/mL) were infused to stomach of the nude mice at an OEA-dose of 5 mg/kg. At 0 h, 1 h, 2 h, 4 h, 6 h, 12 h, and 24 h post-injection, the mice were anesthetized and imaged in vivo with the Maestro imaging system (Cambridge Research & Instrumentation). After 24 h, the mice were sacrificed, and the brain and the major organs (liver, kidney, lung, spleen, and heart) were excised, followed by washing the surface with 0.9% NaCl for fluorescence intensity measurement.

### Animals

The experimental protocols were approved by the Animal Care and Use Committee of Medical College of Xiamen University in compliance with the NIH Guide for the Care and Use of Laboratory Animals (NIH Publications No. 80–23). The male BALB/C nude mice (16–20 g), male Kunming mice (18–22 g) and male Sprague–Dawley (SD) rats (260–280 g) were purchased from Beijing Vital River Experimental Animal Co. (Beijing, China) and housed under a 12/12 h dark/light cycle in specific pathogen-free (SPF) conditions. The animals were fasted without food deprivation for 12 h before the MCAO procedure was performed.

### Drugs administration

Drugs were dissolved in saline with ultrasonic breaking. Drugs (10 mg/kg, *ig*) were administered once in mice/nude mice at the time of redispersion. For rats, drugs (10 mg/kg, *ig*) were administered once daily for 14 consecutive days after ischemia.

### Preparation of the focal cerebral ischemia model

Focal cerebral ischemia was induced by middle cerebral artery occlusion (MCAO) in both adult male Kunming mice and adult male Sprague–Dawley (SD) rats, as previously described [9, 11]. In brief, animals were anesthetized with chloral hydrate (400 mg/kg, ip). The 6-0 (mice) or 4-0 (rats) silicon rubber-coated nylon monofilament was inserted into the right internal carotid artery (ICA) through the external carotid stump, and past the ECA/ICA bifurcation to occlude the origin of the middle cerebral artery (MCA) at the junction of the circle of Willis. The monofilament was kept in place for 90 min (mice) or 120 min (rats) and then withdrawn. Sham-operated animals were treated with an identical surgery except that the intraluminal filament was not inserted. Throughout the procedure, body temperature was maintained at 37 ± 0.5 °C. Animals were excluded if hemorrhage was found in the brain slices or at the base of the circle of Willis during postmortem examination.

### Measurement of infarct volume

Focal cerebral ischemia was induced by MCAO [9]. At 24 h after MCAO, the mice were decapitated, and the brains were removed rapidly and cut into five 2-mm thick coronal sections, which were then stained with standard 2% 2,3,5-triphenyltetrazolium chloride (TTC) at 37 °C for 10 min followed by overnight immersion in 10% formalin. Images of the stained brain sections were captured using a digital camera (FinePix S602 Zoom). The infarct area on each TTC-stained section was measured with Image Tool 2.0 software (University of Texas Health Science Center) and calculated as the infarct area thickness (2 mm). The volume of the infarct is: V_real_ = V_measured_/(V_ipsi_/V_contra_). To determine the extent of ipsilateral oedema, the percentage increase in the ischemic hemisphere volume was calculated by Eq. (2):

2 $${\text{Ipsilateral oedema degree}}(\%) = ({{\text{ipsilateral volume}} - {\text{contralateral volume}}})/{\text{contralateral volume}} \times 100{{\%}}$$

### Evaluation of neurological deficit

At 24 h after MCAO, we tested all the mice/rats for neurological function using an established method, and we eliminated the mice/rats without neurological impairment. Neurological scores were defined as follows: 0, no deficit; (1) flexion of the contralateral forelimb upon lifting the entire animal by the tail; (2) decrease in thrust towards the contralateral plane; and (3) circling to the contralateral side. Additionally, at 3 days, 7 days, and 14 days after reperfusion, rats (n = 6 rats for sham group, and n = 8 rats for each ischemia group receiving different formations) were evaluated neurologically by a single examiner who was blinded to the animal groups.

### Balance beam walking test

Motor coordination and balance were assessed by measuring the ability of rats to traverse a 1 m long beam with a width of 2.5 cm. The beam was placed 50 cm above the floor, with one end mounted on a narrow support and the other end attached to the rat’s home cage. Rats were pre-train for 3 consecutive days (3 trails a day) until they could walk through the beam stably before surgery. At 3 days, 7 days, and 14 days after reperfusion, the score of each rat traversing the balance beam is as follows: (0) if rat traverses the balance beam smoothly without foot-slip of the hind limb; (1) if rat traverses the beam with more than one foot-slip, but less than 50% foot-slips of the hind limb; (2) if rat traverses the beam with more than 50% foot-slips of the hind limb; (3) if rats traverses the balance beam reluctantly, but the hind limbs cannot help move forward; (4) if the rat is unable to move forward, but able to balance on the beam; (5) if rat is unable to stay on the beam. The mean score was calculated by three individual tests.

### Grip strength test

At 3 days, 7 days, and 14 days after reperfusion, the grip test is used to evaluate the muscle strength of rat limbs. Gently place the rat on the grip plate. After the animal is firmly grasped, pull the rat tail backwards to release the claw. The maximum grip of each animal will be recorded automatically. The mean value was calculated by three individual tests.

### Morris water maze task

Morris water maze (MWM) task was begun from day 15 to day 20 after cerebral ischemia. The protocol followed the previous report [11]. Acquisition training consisted of 5 days of conditioning with four trails per day from day 15 to 19. For each trail, the rat was placed in the water at one of the four starting points (north, south, east or west) and allowed to swim for a maximum of 90 s. If the rat found the platform, it was allowed to remain on it for 15 s. If the rat cannot find the hidden platform within 90 s, it would be guided to the hidden platform. The swimming speed and escape latency of finding the hidden platform were recorded. On day 20, the platform was removed and rats were given one 90-s retention probe test. We recoded the swimming traces of the rats by a video camera with a computer via an image analyzer. The time spent in the targeted quadrant and the number of times each animal crossed the position where the platform had been previously located were also measured by the analyser.

### Immunofluorescence staining and cell counting

Mice (24 h after reperfusion) and rats (21 days after reperfusion) were anesthetized with chloral hydrate and perfused transcardially with ice-cold saline followed by perfusion with 4% paraformaldehyde. The brains were removed and dehydration with a 10%, 20%, 30% sucrose gradient and then coronal sectioned (30 μm) using a vibrating microtome (Leica, Wetzlar, Germany). The sections were incubated in PBS containing 0.5% Triton X-100 and 10% normal goat serum for 1 h at room temperature, following by incubation with rabbit polyclonal NeuN (1:500; Abcam, Cambridge, UK) and rabbit polyclonal Iba1 (1:500; Wako, Osaka, Japan) at 4 °C overnight. After several PBS rinses, sections were incubated with Alexa Fluor 594 donkey anti-rabbit IgG (1:500; Invitrogen, Carlsbad, CA, USA) or Alexa Fluor 488 donkey anti-rabbit IgG (1:500; Invitrogen, Carlsbad, CA, USA). The number of NeuN or Iba1 positive cells was analysed by fluorescence confocal microscopy (EX61, Olympus, Tokyo, Japan). The positive cells were counted in three randomly chosen squares of identical size (460 × 460 μm) located in the cortical penumbra or hippocampal CA1 area.

### Toluidine blue staining

The 30-μm section of mice and rats were stained with 1% toluidine blue in PBS for 20 min. After rinsing with double distilled water, they were dehydrated with alcohol and mounted. Representative images of mouse brain slices were obtained using a BX53 Olympus microscope (BX53, Olympus, Tokyo, Japan), and rats brain sections were obtained using a PreciPoint M8 microscope (M8, PreciPoint, Freising, Germany).

### Statistical analysis

The statistical significance of treatment outcomes was assessed using one-way analysis of variance (ANOVA) for the differences within treatments followed by Tukey’s post hoc test (Prism 5 for windows, GraphPad Software Inc., USA); P < 0.05 was considered statistically significant in all analyses (95% confidence level).

## Results and discussion

### Preparation of OEA–SPC Nanoparticles (OEA–SPC NPs)

Different from other drug delivery systems, a key feature of the SPC-based nanoparticles is the preparation of drug-SPC complex via forming hydrogen bond between drug and SPC molecules, which would greatly improve the pharmaceutical properties of the drug. To prepare the OEA–SPC complex, OEA and SPC were codissolved in tetrahydrofuran and stirred for 10 h at 40 °C. Perhaps owing to the complexity of the drug used in the system, which group that could form hydrogen bond with the phosphatide is still not fully understood [30, 31]. In this work, XRD, FT-IR, and H^1^NMR were employed to confirm the existence of hydrogen bonds and the groups forming them.

**Fig. 1:**
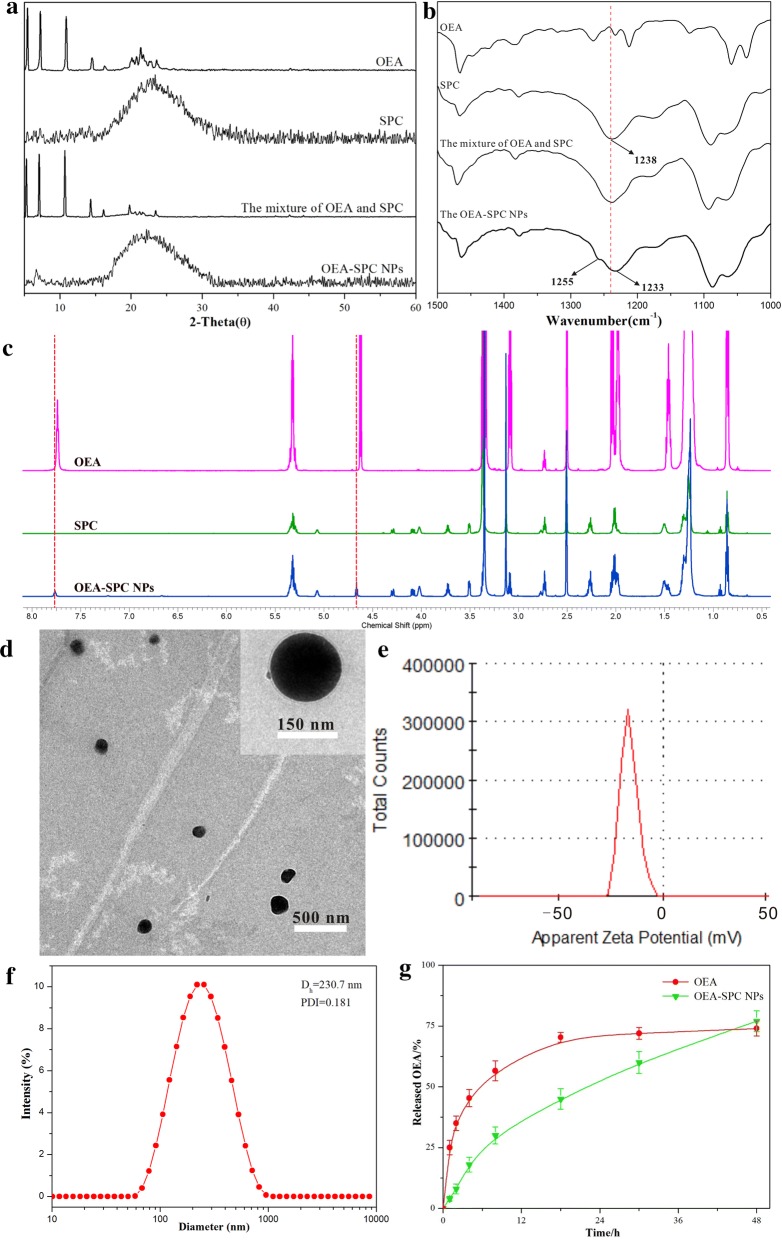
XRD patterns (**a**), FT-IR spectra (**b**) and H^1^NMR spectra (**c**) of OEA, SPC, the mixture of OEA and SPC, and OEA–SPC NPs. TEM image (**d**), zeta potential (**e**), and size distribution (**f**) of OEA–SPC NPs. **g** The in vitro drug release of OEA and OEA–SPC NPs. The ratio of OEA to SPC was the same in the mixture and the OEA–SPC NPs

Firstly, the X-ray diffraction was used to detect the form of OEA within the OEA–SPC NPs (Fig. 1a). The XRD pattern of bulk OEA exhibited many sharp peaks, suggesting its high crystallinity. However, only one weak and broad peak could be observed in the pattern of SPC, illustrating its semicrystalline. As to the mixture of OEA and SPC, the pattern was just similar to that of OEA. This stated that the OEA maintained the same polymorph in the mixture. However, in the XRD pattern of OEA–SPC NPs, almost all of the sharp peaks of OEA disappeared and only the broad peak ascribed to SPC remained unchanged. The results nicely suggested that the preparation process had totally changed the growth kinetics of OEA and the crystalline OEA was existed in the amorphous state within the OEA–SPC NPs. The reason was that the hydrogen bonds might have formed between OEA and SPC molecules.

Then, FT-IR was employed to confirm the groups that formed hydrogen bonds. In the FT-IR spectra of SPC, the absorption peak at 1238 cm^−1^ was ascribed to the aliphatic phosphate P = O stretching vibration, which remained unchanged in the spectra of the mixture (Fig. 1b) Nevertheless, the peak was obviously blueshifted to 1233 cm^−1^, and a shoulder peak emerged at 1255 cm^−1^. The results indicated that the aliphatic phosphate P = O was affected by other new bonds in the OEA–SPC NPs compared with in the mixture. Since none of covalent bonds were newly formed in the OEA–SPC NPs, the influence can only come from the newly formed hydrogen bonds between the aliphatic phosphate P = O and OEA molecules.

When forming hydrogen bonds, the hydrogen atoms would be affected and their chemical shift would make a difference. Hence, the H^1^NMR was used to further search the hydrogen atoms included in the hydrogen bonds. As shown in Fig. 1c, the peak at 7.72 ppm (ascribed to the –NH– of OEA) and the peak at 4.62 ppm (ascribed to the –OH of OEA) appeared to obviously shift to the higher field in the OEA–SPC NPs. The results indicated that the two hydrogen atoms were simultaneously affected by the hydrogen bonds. Combined with the results of XRD, FT-IR and H^1^NMR, it is suggested that the aliphatic phosphate “P = O” could form hydrogen bonds with the “-NH-” or “-OH” of OEA, and thus change the form of OEA in the NPs. Since one aliphatic phosphate “P = O” could only offer a lone pair, “–NH–” and “–OH” of OEA might combine with two SPC molecules simultaneously (Additional file 1: Formula S1).

**Scheme 1:**
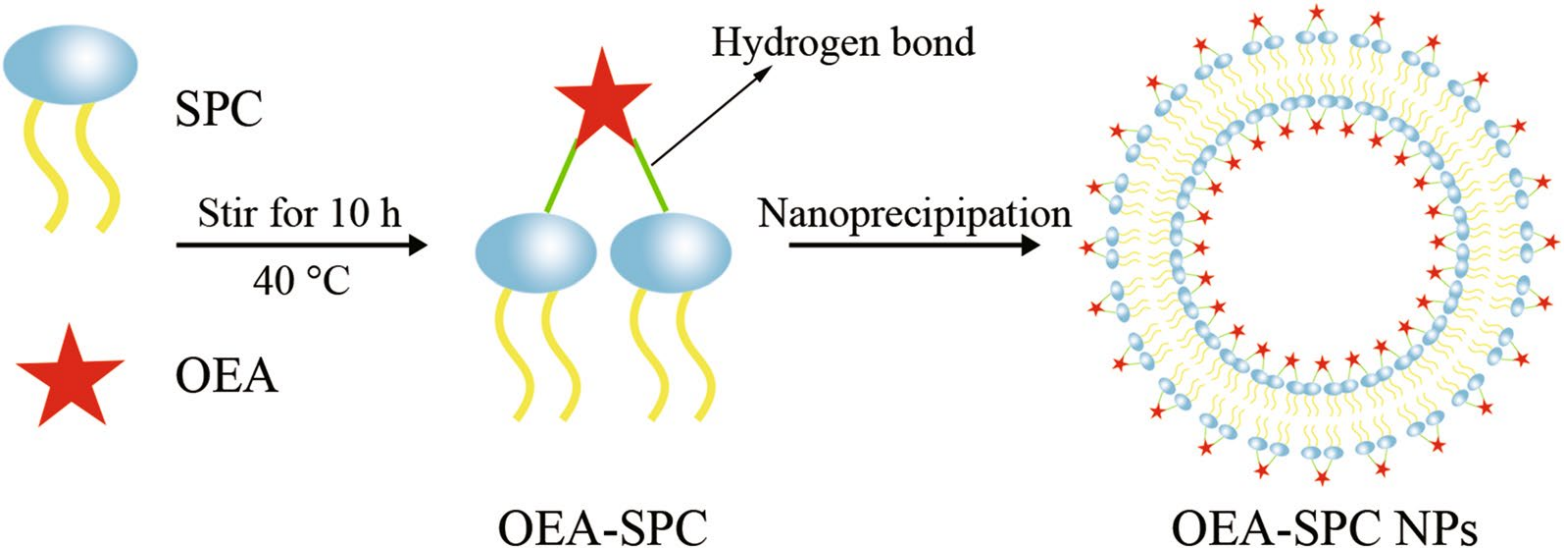
Illustration of the preparation process and the structure of the OEA–SPC and the OEA–SPC NPs

The OEA–SPC NPs were prepared via nanoprecipitation technique, which was based on the amphipathicity of SPC [32, 33]. Briefly, the OEA–SPC complex was dissolved in dichloromethane and diffused toward the continuous phase at a certain, slow speed. Then the system turned into an O/W suspension. With the volatilization of the dichloromethane, a dramatic decrease in the interfacial tension took place, leading to the progressively smaller droplet size. After the evaporation of the organic phase, the system came to a total water environment. Owing to the hydrophobicity of the “tails” of SPC, they would assemble together to keep away from the water environment. Hence, the liposomes loaded with OEA were successfully prepared with a structure of lipid bilayer (Scheme 1).

The characters of the nanoparticles, such as the size, the PDI, the zeta potential, play a paramount role on the fate of a drug delivery system. Hence, to prepare OEA–SPC NPs with better pharmaceutical properties, the characters of OEA–SPC NPs with different drug loading were analyzed. As shown in Table 1, the drug loading of the OEA–SPC NPs increased with the ratio of OEA to SPC. However, the other pharmaceutical properties, such as PDI, size, and zeta potential, became poorer. Especially when the ratio increased from 1:5 to 1:4, the size increased dramatically. The reason might be that the excess bonding with OEA would change the hydrophilia of the “head”, which played a crucial role in the preparation process. As a result, no liposomes were obtained when the ratio came to 1:3 (Table 1).

**Table 1:** The drug loading, zeta potential, size and PDI of OEA–SPC NPs

The ratio of OEA to SPC (w/w)	Drug loading (wt%)	PDI	Zeta potential (mv)	Size (d)
1:3	–	–	–	–
1:4	19.7 ± 1.8	0.224 ± 0.030	− 13.4 ± 1.3	330.8 ± 21.6
1:5	18.3 ± 1.3	0.181 ± 0.019	− 16.8 ± 0.9	230.7 ± 7.9
1:7	13.3 ± 0.6	0.173 ± 0.016	− 19.6 ± 0.7	215.4 ± 7.8
1:10	10.9 ± 0.4	0.170 ± 0.018	− 20.5 ± 0.7	223.4 ± 8.2

Values are mean ± SD, n = 3

Hence the ratio 1:5 was chosen as the optimal condition and used for the following evaluation experiments. Then, photographs of a zeta potential distribution and size distribution of the OEA–SPC NPs are depicted in Fig. 1e, f, respectively. Possessing a drug loading of 18.3 ± 1.3%, a size of 230.7 ± 7.9 nm, a zeta potential of − 16.8 ± 0.9 mv, and a PDI of 0.181 ± 0.019, the OEA–SPC NPs were identified to greatly improve the solubility of OEA, which would substantially increase its bioavailability and make it suitable for oral administration. TEM images (Fig. 1d) exhibited more directly that the OEA–SPC NPs possessed the shape of a relatively integrated sphere, with fairly uniform size and well-distributed character dispersed in water environment.

### In vitro drug release study

The hydrogen bonds between OEA and SPC, plus the lipid-bilayer architecture allow the sustained release of OEA. The in vitro release studies of the OEA–SPC NPs were performed using a dialysis technique, alongside with the bulk OEA powers. All samples were assayed by liquid chromatography-mass spectrometry (LC–MS). The release profiles of OEA are shown in Fig. 1g. With a release of about 45% at 4 h, the burst release of bulk OEA was serious. At the second half time, the release profile was stable at 70%, indicating its incomplete release. This could be owing to the poor water solubility of OEA. As comparison, the OEA–SPC NPs exhibited a remarkably prolonged release profile over the course of experiment. Above all, the release of OEA from OEA–SPC NPs exceeded that from bulk OEA at the last sample time. The reason might be that the OEA–SPC NPs were suspended well in the phosphate buffer solution, which dramatically increased the surface area and hence improved the drug release.

### Biodistribution

**Fig. 2:**
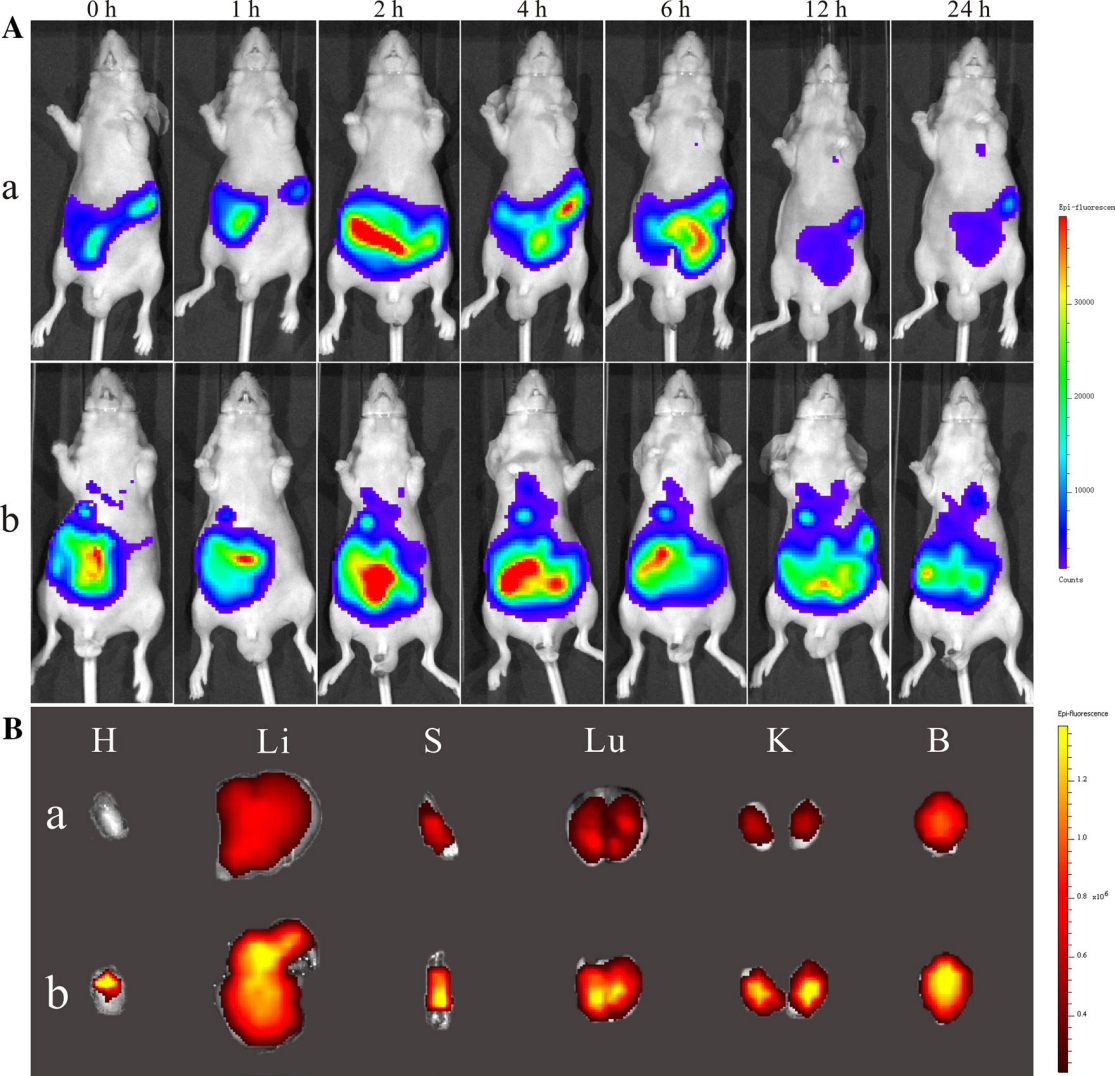
**A** The in vivo biodistribution of the OEA-Cy 5.5 (a) and OEA (-Cy 5.5)-SPC NPs (b) in nude mice receiving intragastrical administration of the indicated formulation. **B** Ex vivo fluorescence imaging of the brain and normal tissues harvested from the euthanized nude mice. The images were taken 24 h after the intragastrical administration of the indicated formulations. H, Li, Lu, K, S, and B represent heart, liver, lung, kidney, spleen, and brain, respectively

The biggest weakness of bulk OEA lay in its lower bioavailability caused by the poor water solubility. To visually exhibit the drug absorption of OEA in vivo, the biodistribution of OEA–SPC NPs, as well as bulk OEA was investigated. To facilitate the observation of OEA in vivo, OEA was labelled with Cy 5.5 at equivalent concentration. Briefly, the nude mice were treated with OEA (-Cy5.5)-SPC NPs or OEA-Cy5.5 via intragastric administration at equivalent concentration, and fluorescent images of the mice were taken at different time intervals to evaluate their biodistribution. As depicted in Fig. 2A, the fluorescent signals emerged the shape of intestinal canal after the administration of OEA-Cy5.5, indicating that the OEA-Cy5.5 just stayed inside and moved along the alimentary canal, and a majority of the OEA-Cy5.5 could not be absorbed. As comparison, the OEA (Cy5.5)-SPC NPs could obviously extend outside of the alimentary canal, suggesting that they were absorbed into the blood circulation system. Another meaningful improvement is that the OEA (-Cy5.5)-SPC NPs could greatly prolong their duration in vivo. At the sample time of 24 h, the mouse treated with OEA (-Cy5.5)-SPC NPs still fluoresced a very intense signal, which was significantly higher than that from the mouse treated with OEA-Cy5.5. The reason were as follows: on one hand, the unabsorbed OEA-Cy5.5 was excreted out, leading to the sharply decrease of fluorescence signal. On the other, owing to the controlled release of OEA (-Cy5.5)-SPC NPs, OEA could be sustained release from the NPs, which would keep the fluorescence signal at a relatively high level.

After 24 h, the mice were sacrificed and the brain as well as the normal tissues were isolated for analysis (Fig. 2B).The fluorescence intensity from the tissues of the mouse treated with OEA (-Cy5.5)-SPC NPs was significantly higher than the OEA-Cy5.5 group, validating that OEA (-Cy5.5)-SPC NPs offered the OEA a more enhanced bioavailability. Especially in the brain, the fluorescence intensity of the mouse treated with the OEA (-Cy5.5)-SPC NPs was three time as much as that of the OEA-Cy5.5 group, which would leading to a highly efficient anti-stroke treatment (Additional file 1: Figure S1). In addition to the better absorption, we speculate that the combination of OEA with SPC was speculated to help penetrate the blood–brain barrier and hence increase their accumulation in brain, which might further enhance their neuroprotective effect.

### The evaluation of neuroprotective effects in mice

**Fig. 3:**
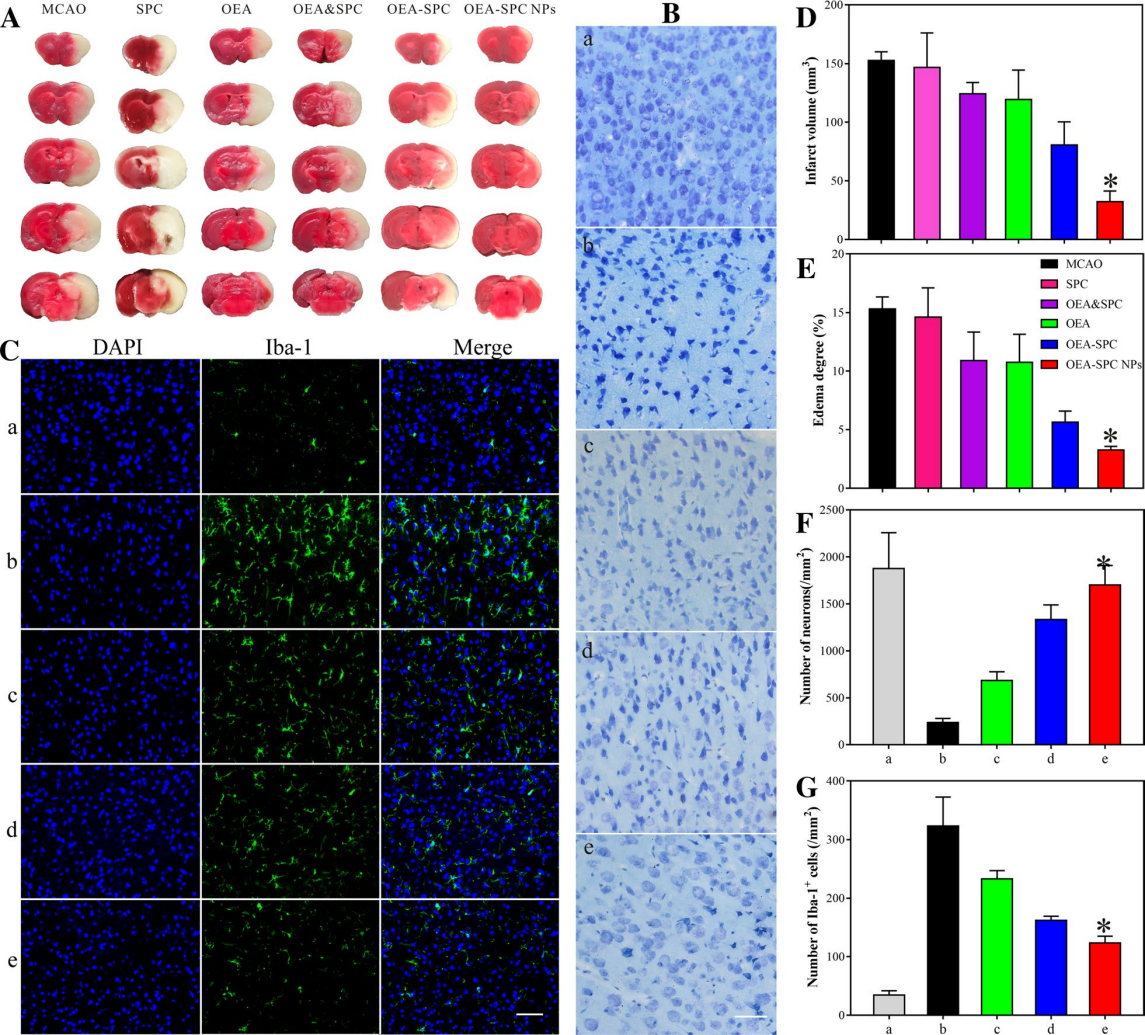
Therapeutic effects of the indicated formulations (0.9% NaCl (Sham), SPC, OEA and SPC, OEA, OEA–SPC, and OEA–SPC NPs, [OEA] = 10.0 mg/kg, [SPC] = 44.6 mg/kg) on cerebral damage after MCAO in mice. The infarct volume (**A**, **D**), oedema degree (**E**), number of neurons (**B**, **F**) and number of Iba-1^+^ cells (**C**, **G**) were detected at 24 h after reperfusion in mice. OEA and SPC: The mixture of OEA and SPC; OEA–SPC: The OEA–SPC complex. “a–e” in **B**, **C**, **F**, and **G** represent Sham, MCAO, OEA, OEA–SPC, and OEA–SPC NPs, respectively. Bar in figure B is 100 µm, and bar in figure C is 100 µm. Data are expressed as mean ± SD. *P < 0.01 VS. MCAO group

To assess the in vivo neuroprotective effects of the OEA–SPC NPs on ischemic cerebral injury, a systematic experiment were performed on mice and rats. Firstly, the cerebral infarct volume is one of the most important evaluation indicators, which could visualized reflect the state of stroke. The TTC-stained brain slices revealed the cerebral infarct volume of the MCAO models treated with different formations (Fig. 3A). Since the SPC was reported to maintain potential neuroprotective effects, the SPC and SPC and OEA groups were added as control. As shown in Fig. 3A and the statistical data (Fig. 3D), the mice treated with SPC came into the similar cerebral infarct volume to that of the MCAO group, indicating that SPC did not have significant effect on improving the cerebral infarct. The same conclusion could also be reached by comparing the OEA group to the SPC and OEA group. Although the cerebral infarct volume of OEA group was reduced from 176.1 ± 4.2 to 132.4 ± 13.8 cm^3^ compared to the MCAO group, there was no significant difference between the OEA and MCAO groups at the dosage of 10 mg/kg (P > 0.05), which was in according with our previous study [9–11]. Above all, a dramatic improvement had emerged when OEA was associated with SPC via hydrogen bonds (the OEA–SPC complex group). The reason was that the hydrogen bonds had totally changed the form of OEA and increased the water solubility. When OEA–SPC NPs was prepared, there was a further improvement. The cerebral infarct volume was decreased to be 33.3 ± 4.7 cm^3^, which only accounted for 25.2% of that of OEA group and 18.9% of the MCAO group. This highly pronounced therapeutic effect could be owing to the protection of OEA by the nanostructure plus the hydrogen bonds, which could greatly increase the bioavailability of OEA. A pathological phenomena of ischemic cerebral is cerebral oedema, which could also be observed that the volume of the ischemic hemisphere was significantly increased compared with the normal hemisphere (Fig. 3A). According to the statistical data of cerebral oedema (Fig. 3E), the ipsilateral oedema of the OEA–SPC NPs was about 3.3%, which was reduced by 78.4% compared with 15.3% of the MCAO group and 69.2% compared with 10.7% of the OEA group. The result indicated that the OEA–SPC NPs possessed highly efficient improvement to the cerebral infarct volume and the subsequent cerebral oedema.

The intractable sequelae of stroke lies in the injury of neuron, which was really hard to recover. The neuron damage of the mice treated with different formations were evaluated with toluidine blue stain (Fig. 3B). The normal neurons were oval shaped with wathet or translucent blue color, just as shown in Fig. 3B-a. When the neurons were injured, the chromatin were pyknotic, leading to deepened color as well as shrunken and irregular shape (Fig. 3B-b). Compared with almost all the neurons injured of MCAO group, the terrible situation was largely improved via the administration of OEA–SPC NPs. Approximately 75% neurons were well protected and maintained their normal appearance (Fig. 3F). The result stated that the OEA–SPC NPs could perform well in the protection of the neurons from ischemic cerebral injury.

To a stroke patient, the ischemic reperfusion brain injury is the primary cause of the serious sequelae, which was mainly induced by the inflammation of reperfusion. Iba-1 is a unique marker of microglial cell, which plays a paramount role in the brain inflammation. Hence, the quantity of expressed Iba-1 was used to evaluate the inflammation of reperfusion. As depicted in Fig. 3C-a, there are less number of Iba1 positive cells which have small cell body in the sham group. As comparison, the Iba1 positive cells of MCAO group exhibited larger cell body and number, which could be reduced almost to the normal level by the administration of OEA–SPC NPs (Fig. 3C-e, G). These results indicated that the OEA–SPC NPs could greatly alleviate the inflammation induced by ischemic reperfusion, and hence, provide significant neuroprotective effects.

### The evaluation of neuroprotective effects in rats

Interestingly, we found the mice mortality rate after MCAO at convalescence is too high. However, the mortality rate of rats is relatively low. Therefore, we used rats to evaluate the neuroprotective effect at the chronic phase of stroke. Since the neurons of the stroke patients were damaged, their behavior coordination might terribly impaired, which possibly caused hemiplegia. The effect of the formations mentioned beforehand on the rats’ behavior were continuingly investigated. The bederson score, beam walking score, and the grip strength were assessed at 3 days, 7 days, and 14 days after MCAO. Compared with the sham group, the rats of the MCAO group made almost the poorest scores in all the three indexes at 3 days. Although the score were higher in the following two evaluation, they still kept at a very low level, suggesting their destroyed motor ability. When the MCAO rats were treated with different formations, their performance become better to different extents. The OEA–SPC NPs still obtained the most improvement, which was in according with our previous test on mice.

**Fig. 4:**
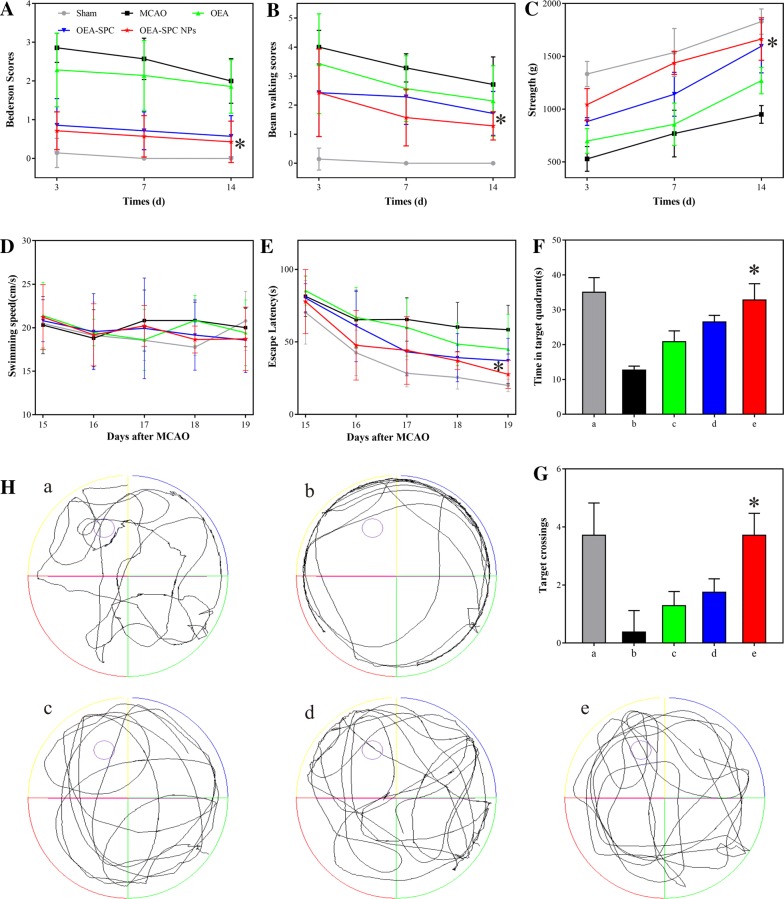
The evaluation of the behavior ability. The Bederson scores (**A**), beam walking scores (**B**), and holding power (**C**) of rats treated with the indicated formations (0.9% NaCl (Sham), SPC, OEA and SPC, OEA, OEA–SPC, and OEA–SPC NPs, [OEA] = 10.0 mg/kg) at 3 days, 7 days, and 14 days after reperfusion. The effect of the formations on MCAO-induced spatial cognitive deficits. The swimming speed (**D**), and escape latency (**E**) in the hidden platform trails. The time spent in the target quadrant (**F**) the number of target platform crossings (**G**) and swimming trace (**H**) in the spatial probe trials. The legend of (B-E) were the same as that in **A**. “a–e” in **F**–**H** represent Sham, MCAO, OEA, OEA–SPC, and OEA–SPC NPs, respectively. Data are expressed as mean ± SD. n = 10–12 rats per group. *P < 0.01 vs. MCAO group

Learning and memory are the important computational strategies of the brain, which might also be impaired by ischemia reperfusion. To examine the effects of OEA–SPC NPs on spatial learning and memory, rats were exposed to the water maze task after 10 days of OEA–SPC NPs treatment and 7 days of drug withdrawal. Spatial learning was assessed by the time required to find the platform (escape latency), and the memory was assessed by the ability to find the removed platform (Target crossing and Time in target quadrant). Firstly, the precondition for the assessment is the same swimming speed. As shown in Fig. 4D, all the rats swam at a speed of around 20 cm/s, ensuring the fairness and validity of the measurement. Compared with the sham group, the rats of the MCAO group exerted much longer escape latency, which could be markedly decreased via the treatment of OEA–SPC NPs (Fig. 4E). Another stronger evidence for the improvement of the impaired cognitive ability is the significantly decreased escape latency at 19 days compared with that at 15 days. Across the 5-day training period, the escape latency of the OEA–SPC NPs group reduced from 77.83 ± 4.59 to 27.67 ± 3.98 s (64.5% off), whose extent was much greater than the 28.3% of the MCAO group. The data forcefully illustrated that the OEA–SPC NPs treatment effectively improved the spatial learning, which even had no significant differences with the sham group (P > 0.05). At 20 days, the platform was removed, and the traces of rats in the water maze task were recorded (Fig. 4H). The rats treated with OEA–SPC NPs exhibited markedly prolonged time in the target quadrant and increased number of the crossing platform position, compared with the MCAO and OEA group (Fig. 4F, G), indicating their stronger potential memory for the removed platform. Therefore, OEA–SPC NPs greatly ameliorated ischemia-induced spatial memory impairment.

**Fig. 5:**
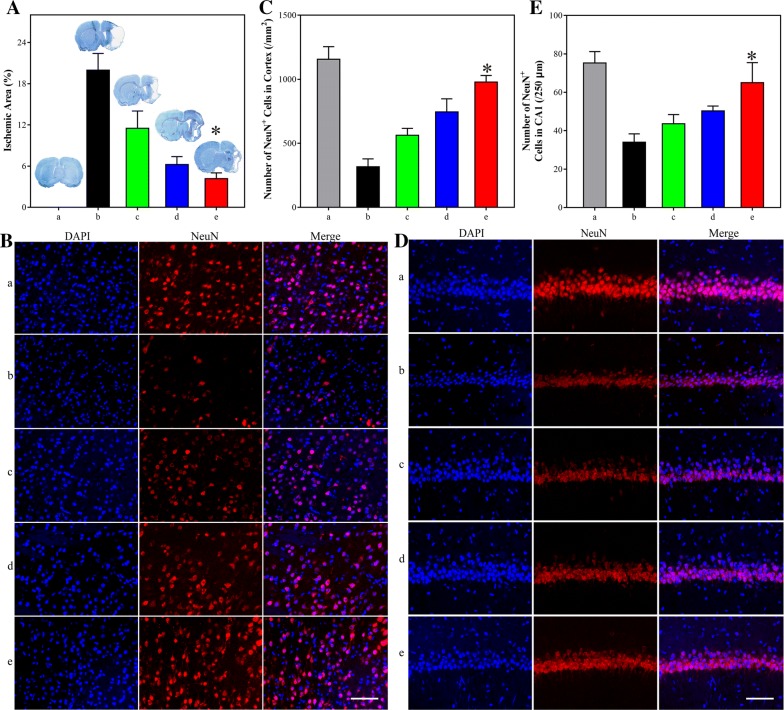
**A** The ischemic area of rats treated with indicated formations (0.9% NaCl (Sham), SPC, OEA and SPC, OEA, OEA–SPC, and OEA–SPC NPs, [OEA] = 10.0 mg/kg). Rats were sacrificed 21 days after reperfusion and immunohistochemistry was performed to identify the phenotype of NeuN-positive cells in cortex (**B**) and hippocampal CA1 (**D**). Quantitative analysis of NeuN + cells in cortex (**C**) and hippocampal CA1 (**E**). “a–e” represent Sham, MCAO, OEA, OEA–SPC, and OEA–SPC NPs, respectively. Data are expressed as mean ± SD. n = 10–12 rats per group. Bar is 100 µm. *P < 0.01 vs. MCAO group

After the Morris water maze test, the rats were sacrificed and the brains were isolated for further analysis. Firstly, toluidine blue stain was employed to exhibit the ischemic area of the brains (Fig. 5A). Compared with the whole brain of the sham-operated rats, about 20% brain missing stated the severe condition of the MCAO rats. Across the 10-days administration of OEA–SPC NPs, the brain was almost fully emerged with only 5% deficiency. The data once again revealed the highly outstanding efficiency of neuroprotection.

To microcosmic observe the condition of the brains, the cerebral cortex were treated with NeuN antibodies to image the functional neuron (Fig. 5B, C). In addition to the dramatically decreased quantity (Fig. 5C), the light color and the irregular revealed that the neuron of the MCAO group were in a terrible condition (Fig. 5B-b). As shown in Fig. 5B-e, C, the OEA–SPC NPs could protect the neuron from the ischemia reperfusion, leading to that the neuron quantity picked up approximately to the level of the sham-operated group (P > 0.05). As mentioned before, the inflammation induced by the reperfusion are greatly pernicious to the neuron. Hence, the inflammations in the cortex were also evaluated via the analysis of the Iba-1 positive cells. As was expected, the inflammation of the rats treated with the OEA–SPC NPs was markedly reduced, compared with that of the MCAO and OEA group (Additional file 1: Figures S2 and S3). Since the OEA–SPC NPs could improve cognitive ability, in which the hippocampal mainly involved, the hippocampal might also obtain protection. Therefore, the neuron condition and the inflammation of hippocampal CA1 were evaluated, simultaneously. Just in according with the result of the cortex, the neuron condition and the inflammation were largely improved (Fig. 5D, E, Additional file 1: Figures S4 and S5).

## Conclusions

In summary, the current study presents a superefficient anti-stroke formation via a simple manufacturing process. The OEA–SPC NPs almost eliminate the adverse effect of ischemic reperfusion. The evaluation indicators including ischemic areas, edema degree, the inflammations, the behaviors, and the cognitive ability were all dramatically improved, which was very close to that of the sham-operated group. These results highlights the feasibility of OEA–SPC NPs for clinic anti-stroke application and might give a voice to thousands of stroke patients in the future. Particularly interestingly, the combination of the NDDS with stroke therapy opens a door for the extended application of NDDS. Other effective but insoluble drug candidates, not only for cancer, but also for stroke or other serious diseases, might achieve another success via combining with the NDDS.

## Additional file

**Additional file 1: Formula S1.** The formal structural formula of the hydrogen bonds. **Figure S1.** Ex vivo fluorescence intensity of brains and normal organs harvested from nude mice intravenously treated with the OEA-Cy 5.5 or OEA(-Cy 5.5)-SPC NPs at 24 h post-injection. **Figure S2.** Iba-1+ cells in cortex. **Figure S3.** Quantitative analysis of Iba-1+ cells in cortex. **Figure S4.** Iba-1+ cells hippocampal CA1. **Figure S5.** Quantitative analysis of Iba-1+ cells hippocampal CA1.
