# Supplementary material for: Integration of phospholipid-complex nanocarrier assembly with endogenous N-oleoylethanolamine for efficient stroke therapy
Source: J Nanobiotechnology. 2019 Jan 19;17:8. doi: 10.1186/s12951-019-0442-x (PMC6339692; doi:10.1186/s12951-019-0442-x)
Supplement: Supplementary file 1 — Additional file 1: Formula S1. The formal structural formula of the hydrogen bonds. Figure S1. Ex vivo fluorescence intensity of brains and normal organs harvested from nude mice intravenously treated with the OEA-Cy 5.5 or OEA(-Cy 5.5)-SPC NPs at 24 h post-injection. Figure S2. Iba-1+ cells in cortex. Figure S3. Quantitative analysis of Iba-1+ cells in cortex. Figure S4. Iba-1+ cells hippocampal CA1. Figure S5. Quantitative analysis of Iba-1+ cells hippocampal CA1. [file 12951_2019_442_MOESM1_ESM.docx]

**Electronic Supplementary Information (ESI)**

**Integration of Phospholipid-complex Nanocarrier Assembly with Endogenous N-oleoylethanolamine for Enormously Effective Stroke Therapy**

Xiangrui Yang, Lanxi Xu, Juan Zhou, Yunlong Ge, Shichao Wu, Junxiong Huang, Ying Li, Maoshu Zhu, Xin Jin, & Lichao Yang

**The file includes**

1. Formula S1
2. Figure S1-5





**Formula S1.** The formal structural formula of the hydrogen bonds.





**Figure S1.** *Ex vivo* fluorescence intensity of brains and normal organs harvested from nude mice intravenously treated with the OEA-Cy 5.5 or OEA(-Cy 5.5)-SPC NPs at 24 h post-injection. Data are presented as mean ± SD (n = 3). ***P < 0.05


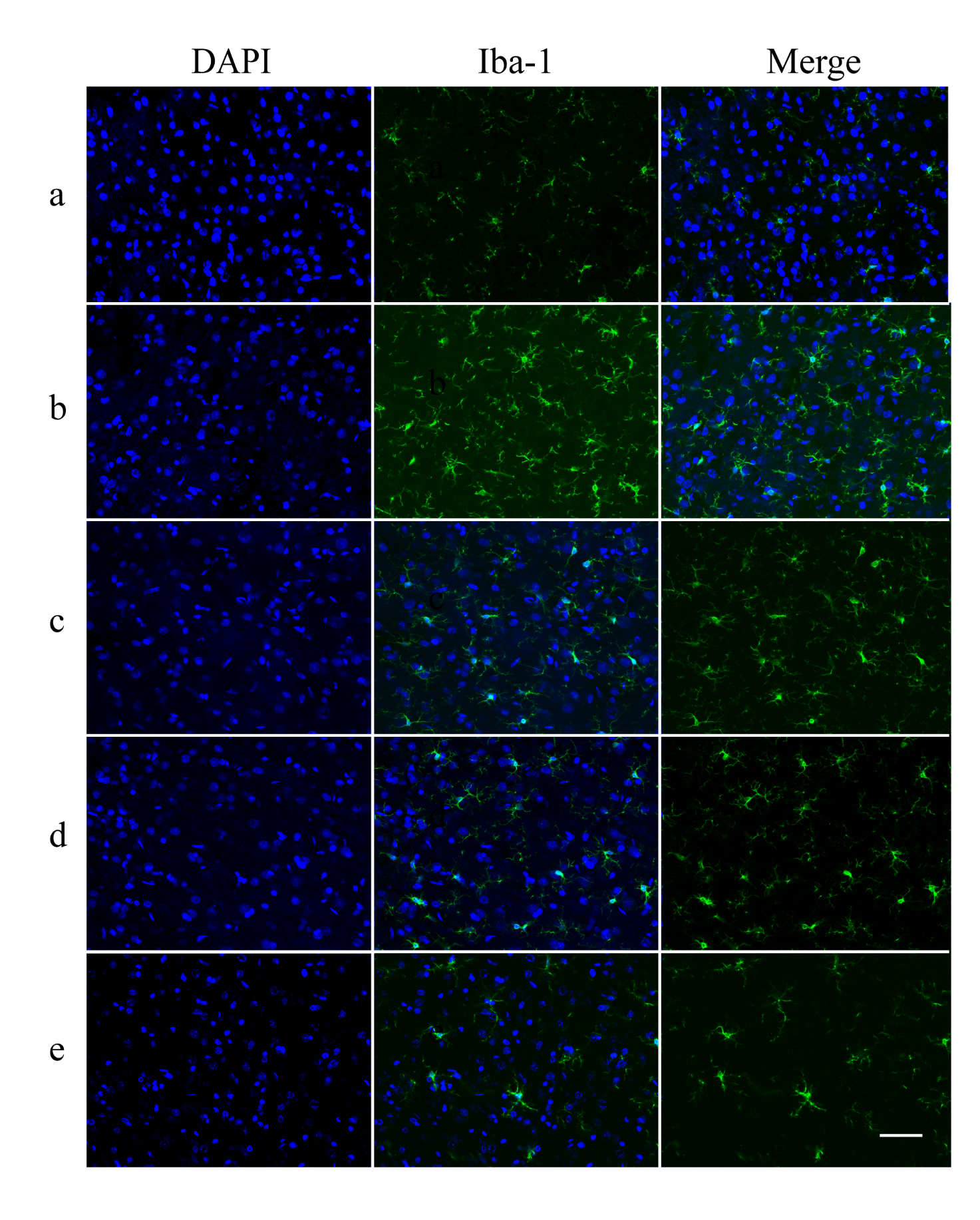


**Figure S2** Iba-1+ cells in cortex. “a, b, c, d, and e” represent Sham, MCAO, OEA, OEA-SPC, and OEA-SPC NPs, respectively. Data are expressed as mean ± SEM. n = 10–12 rats per group. Bar is 100 µm.

**
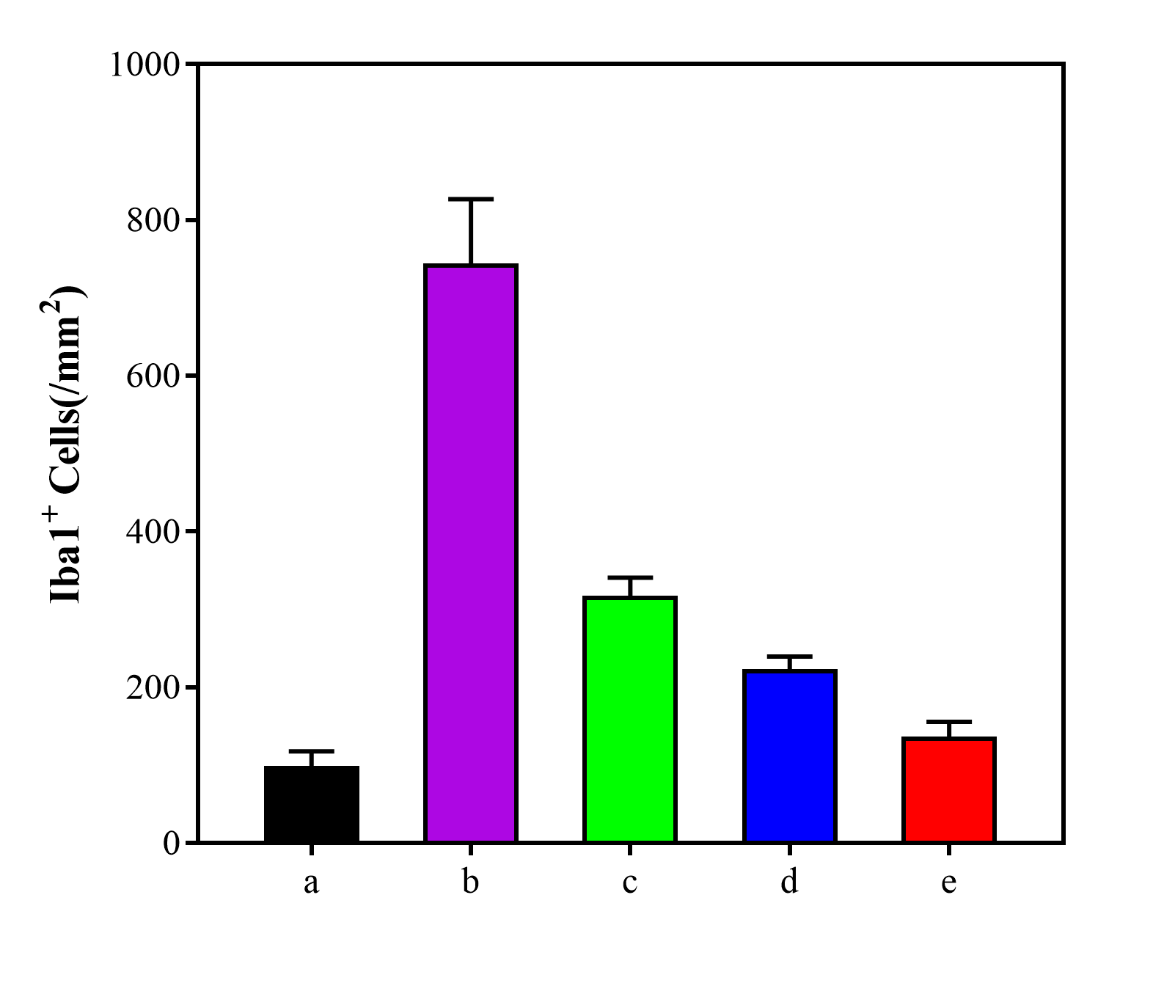
**

**Figure S3** Quantitative analysis of Iba-1+ cells in cortex. “a, b, c, d, and e” represent Sham, MCAO, OEA, OEA-SPC, and OEA-SPC NPs, respectively. Data are expressed as mean ± SD. n = 10–12 rats per group. *P < 0.01 vs. MCAO group.

**
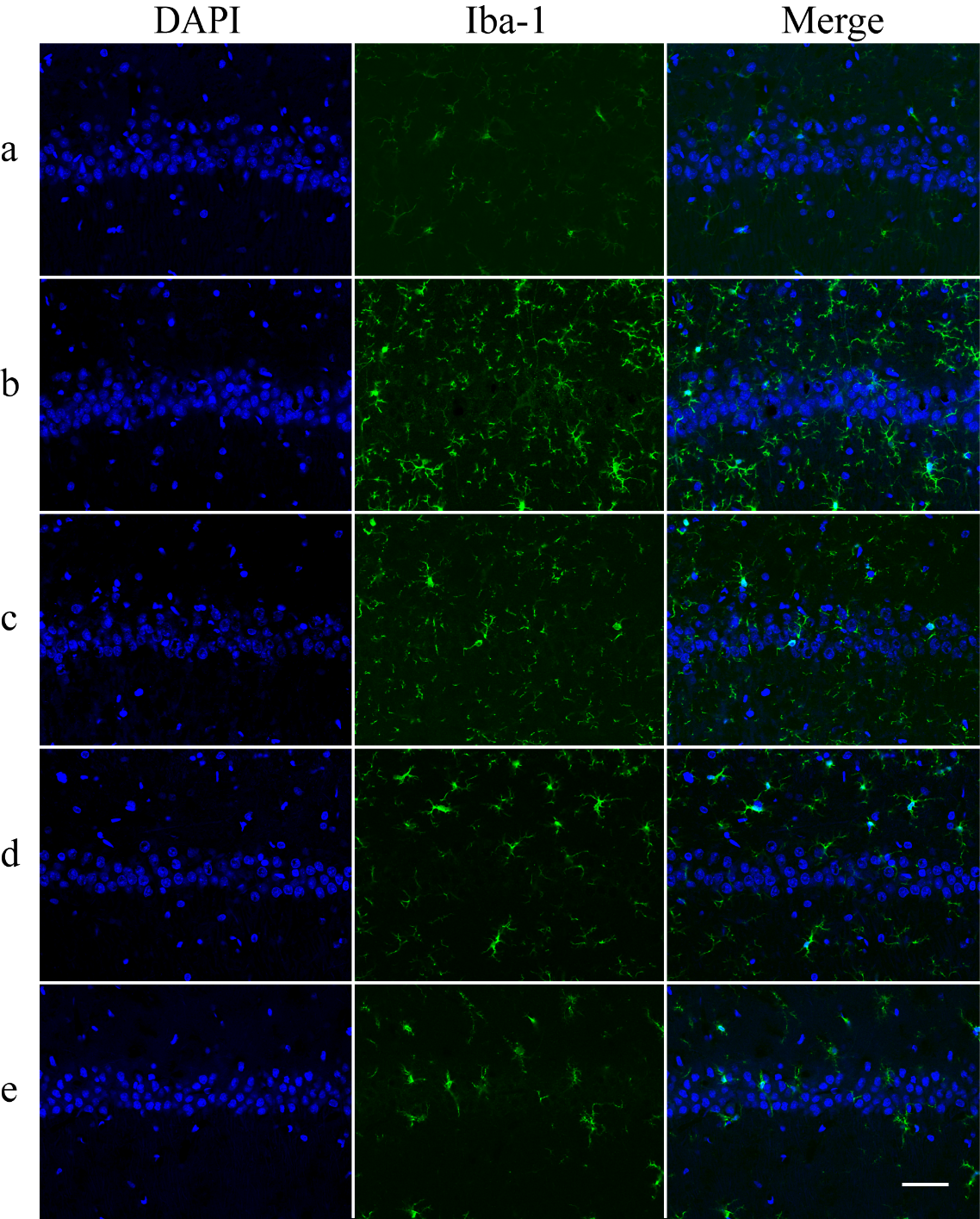
**

**Figure S4** Iba-1+ cells hippocampal CA1. “a, b, c, d, and e” represent Sham, MCAO, OEA, OEA-SPC, and OEA-SPC NPs, respectively. n = 10–12 rats per group. Bar is 100 µm.

**
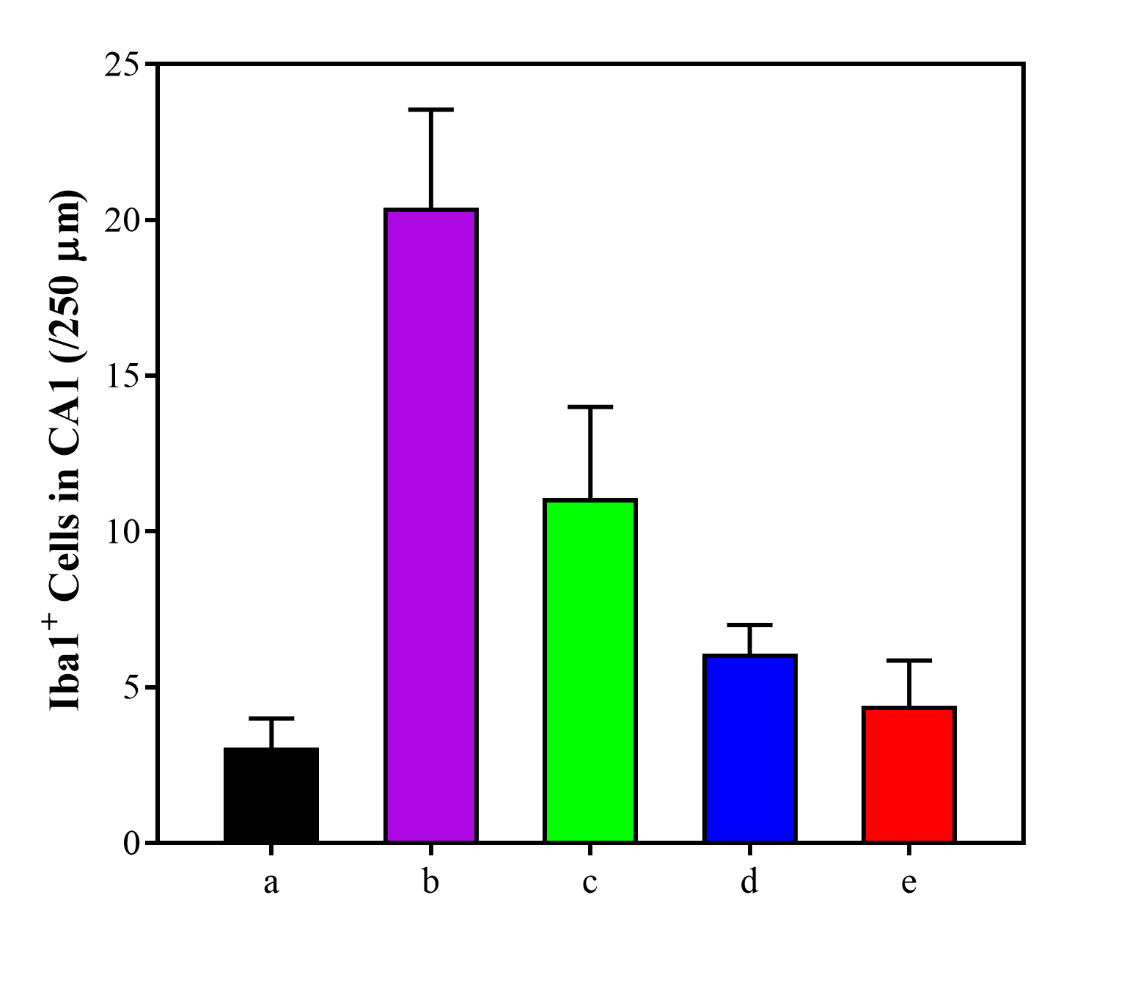
**

**Figure S5** Quantitative analysis of Iba-1+ cells hippocampal CA1. “a, b, c, d, and e” represent Sham, MCAO, OEA, OEA-SPC, and OEA-SPC NPs, respectively. Data are expressed as mean ± SD. n = 10–12 rats per group. *P < 0.01 vs. MCAO group.
